# PyGellermann: a Python tool to generate pseudorandom series for human and non-human animal behavioural experiments

**DOI:** 10.1186/s13104-023-06396-x

**Published:** 2023-07-05

**Authors:** Yannick Jadoul, Diandra Duengen, Andrea Ravignani

**Affiliations:** 1 Comparative Bioacoustics Group, Max Planck Institute for Psycholinguistics, Nijmegen, The Netherlands; 2 Center for Music in the Brain, Department of Clinical Medicine, Aarhus University & The Royal Academy of Music Aarhus/Aalborg, Aarhus, Denmark; 3 Department of Human Neurosciences, Sapienza University of Rome, Rome, Italy

**Keywords:** Animal cognition, Experimental psychology, Randomization, Simple heuristics, Python, Psychometrics, Two-alternative forced-choice, Go/no-go

## Abstract

**Objective:**

Researchers in animal cognition, psychophysics, and experimental psychology need to randomise the presentation order of trials in experimental sessions. In many paradigms, for each trial, one of two responses can be correct, and the trials need to be ordered such that the participant’s responses are a fair assessment of their performance. Specifically, in some cases, especially for low numbers of trials, randomised trial orders need to be excluded if they contain simple patterns which a participant could accidentally match and so succeed at the task without learning.

**Results:**

We present and distribute a simple Python software package and tool to produce pseudorandom sequences following the Gellermann series. This series has been proposed to pre-empt simple heuristics and avoid inflated performance rates via false positive responses. Our tool allows users to choose the sequence length and outputs a .csv file with newly and randomly generated sequences. This allows behavioural researchers to produce, in a few seconds, a pseudorandom sequence for their specific experiment. *PyGellermann* is available at https://github.com/YannickJadoul/PyGellermann.

## Introduction

What is the best way to present experimental stimuli in random order? Two-alternative forced-choice tasks and go/no-go paradigms are common methodologies in human psychological and animal behaviour experiments. These tests require a participant to choose between two options; in the two-alternative forced-choice paradigm these options are two actual stimuli [1, 2], while in the go/no-go paradigm the participant is expected to show a behaviour in response to a positive stimulus (“go”), and to inhibit that behaviour in response to a negative one (“no-go”) [3, 4].

The commonality among all these experiments and paradigms is that in each trial a participant can choose between two actions, of which only one is correct. From an experimenter’s perspective, the question then is how to decide in which order to present these trials? The obvious answer is randomisation: randomised sequences aim at preventing the predictability of a stimulus, and thereby false positive results in the absence of learning. The experimenter randomises, within a sequence, which of the two possible responses—hereafter referred to as A and B—is correct in each trial.

Purely random sequences, however, can accidentally contain some regularities which may be leveraged by participants. This is a potential issue during testing, when the accidental regularities in a sequence match the participant’s cognitive biases and inflate the perceived performance. It also risks hindering learning, if a participant picks up on unintended regularities in presented sequences (e.g., risking to develop a position/colour preference; [5]). Let us consider a random sequence of two types of trials, A and B, in which the number of A/B trials is not balanced 50/50. In this case, an animal sticking to response A (e.g., because of a stimulus or side preference; [2, 6]) will often be able to achieve >50% correct answers within an experimental session. Likewise, a random binary sequence might also feature many alternations of adjacent As and Bs. A participant with a natural tendency to alternate between A and B, will be successful in correctly guessing significantly more than the expected 50%, when the alternating guesses happen to match those of the sequence. The particular alternation strategy adopted by the subject may even result in 70% correct choices by chance [7]. This can be a problem, as the learning criterion—the benchmark to consider a task learned—is often 70% correct choices in a pre-set amount of consecutive sessions [1, 8]. Moreover, when such patterns in random sequences match a participant’s cognitive biases, these trials might incorrectly confirm and reinforce these pre-existing biases rather than advance the learning process. Another common example of these biases is the win-stay, lose-switch strategy witnessed in human psychology.

**Fig. 1:**
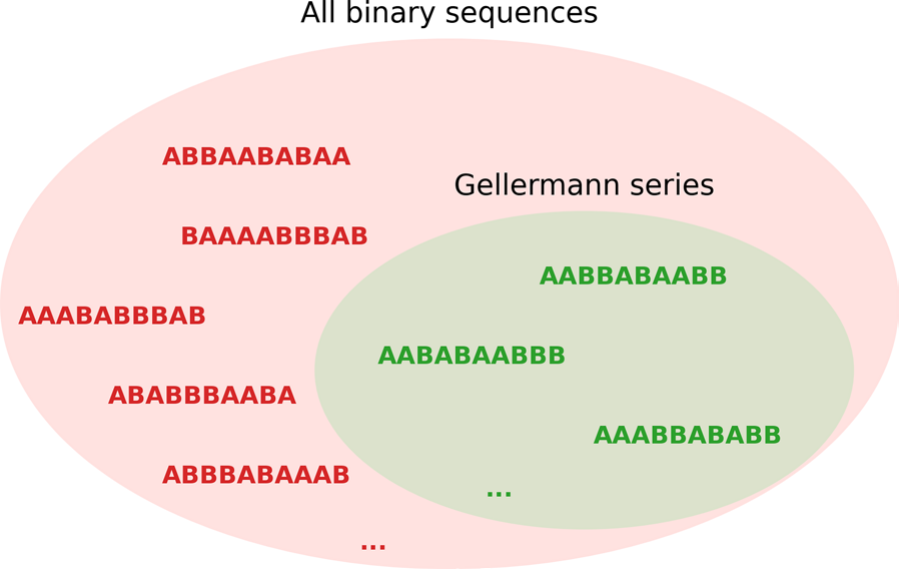
A Venn diagram shows how the set of Gellermann series is a strict subset of all possible binary sequences. Some exemplary sequences (in red) violate some of the criteria put forward by [7]. For instance, in red from top to bottom, the set of Gellermann series does not include the sequences (1) ABBAABABAA because it does not contain an equal number of As and Bs, (2) BAAAABBBAB as it contains 4 As in a row, (3) AAABABBBAB because it has only 1 B and in the first half of the sequence and only 1 A in the second, (4) ABABBBAABA because it contains 6 reversals, and (5) ABBBABAAAB as it provides an 80% correct response rate when responses follow a simple alternation pattern (i.e., ABABABABAB). On the contrary, the sequences AABBABAABB, AABABAABBB, AAABBABABB (in green) fulfill all criteria and are included in the nested set of Gellermann series

In general, building randomised but balanced trial sequences that are immune to simple cognitive biases and ensure that a participant has indeed learned, is not obvious [9, 10]. Several methods exist to circumvent scenarios where, because of an unfortunate pick of presentation order, humans and other animals can accidentally succeed at a task without learning. The most common one is the use of Gellermann series [7], which puts some constraints on the randomization and is often applied when experimental sessions contain relatively few trials (Fig. 1). This is a somewhat paradoxical situation: Mathematically speaking, the introduction of extra constraints increases the predictability of a sequence, which can in turn be exploited by a rational statistical learner [11]. Nevertheless, Gellermann series are a popular heuristic to deal with the potentially irrational cognitive biases in human and non-human animal cognitive experiments, and are finding use in research fields such as psychophysics, neuropsychology, comparative psychology, and animal behaviour [3, 12–16]. The number of trials per session in animal behaviour experiments can be as low as 10 [1, 17, 18], 20 [5], 30 [2, 19, 20], 40 [12], 40 to 60 [15], or 50 to 100 [21].

## Main text

Behavioural researchers are therefore faced with an apparent paradox: they need to create sequences that are as random as possible, but exclude those that would overestimate the experimental participants’ performance by coinciding with simple, non-learning behaviour [22]. The solution typically adopted is to use a Gellermann series, a random sequence which satisfies five criteria to avoid inflating the score of simple psychological or behavioural patterns. However, in existing literature, these sequences are only determined for a fixed sequence length [7], analysed theoretically [23, 24], implemented in programming languages that are not customary used anymore [25], or only partially implemented [26]. To circumvent this problem, and building upon previous work [25], we have developed a Python software package and graphical tool which generates ready-to-use comma-separated values (CSV) files containing Gellermann series. Our tool allows customization of 4 parameters: the length of sequences, the tolerated divergence from 50% chance rate for single and double alternation (see below), the specific string names of the A and B potential choices, and the number of sequences to produce.

Our software package, *PyGellermann*, consists of a Python library and accompanying graphical user interface. Whereas the library allows users to integrate Gellermann series generation into a larger experimental setup, a graphical user interface makes our tool more accessible to researchers who simply want to generate a number of series without writing a single line of code. The advantage of having our tool in Python is that this programming language is broadly used in scientific research, and can also be combined with other tools from the Python scientific ecosystem (e.g., to create sequences of audio stimuli with *thebeat*; [27]). Providing the original Python code has the added advantage that readers can modify it, possibly adapting the original Gellermann criteria [7] to their species of interest, and hence to the particular heuristics that species is prone to [22].

From a mathematical perspective, Gellermann series of length *n* includes 5 constraints (Fig. 1). While the original 5 conditions stated by [7] only applied to length n=10, they can be generalised in a straightforward way to sequences of different length, following [25]. Each series of length *n*:

1. must contain an equal number (= *n*/2) of As and Bs;
2. must contain at most 3 As or Bs in a row;
3. must contain at least 20% (= *n*/5) As and Bs within both the first and last half;
4. must contain at most *n*/2 reversals (A–B or B–A transitions);
5. must provide a correct response rate close to 50% chance^1^ when responses are provided as simple alternation (ABAB...) or double alternation (AABBAA...and ABBAAB...).

**Fig. 2:**
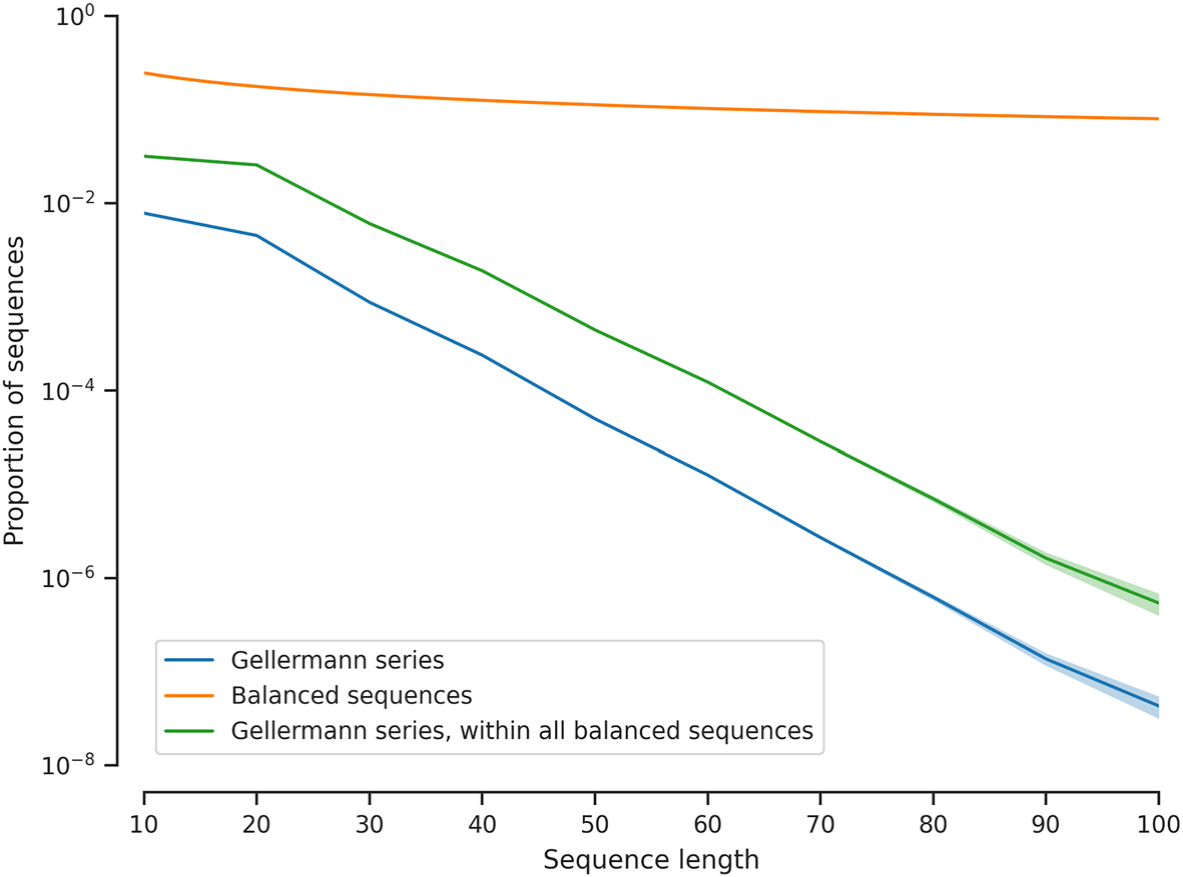
Monte Carlo estimates of the proportion of all binary sequences that meet all five of Gellermann’s criteria, in function of the length of the sequence. Since the proportion drops off exponentially and our implementation generates and tests balanced sequences (i.e., those with an equal number of As and Bs) uniformly at random, generating Gellermann series with many more than 100 elements quickly becomes infeasible

From a computational perspective, our code satisfies these mathematical constraints by repeatedly generating random permutations of a sequence with equal numbers of As and Bs (cfr. condition 1), and checking the other 4 conditions until a valid sequence is found. This procedure becomes computationally expensive for large sequences of $n > 100$ (generating one sequence of length 100 takes about 30 to 60 s, but the time required grows exponentially with *n*; see Fig. 2). However, we are not aware of a surefire way of generating uniformly random Gellermann series more efficiently, and today’s computational power ensures that this procedure is convenient in contexts similar to studies that have used Gellermann series in the past. In addition, because of the law of large numbers, longer truly random binary sequences are more probable to have properties close to the above criteria (i.e., constraints 1, 3, 4, and 5; constraint 2 can be manually enforced after). Thus, for longer sequences, explicitly generating Gellermann series becomes arguably less important.

**Fig. 3:**
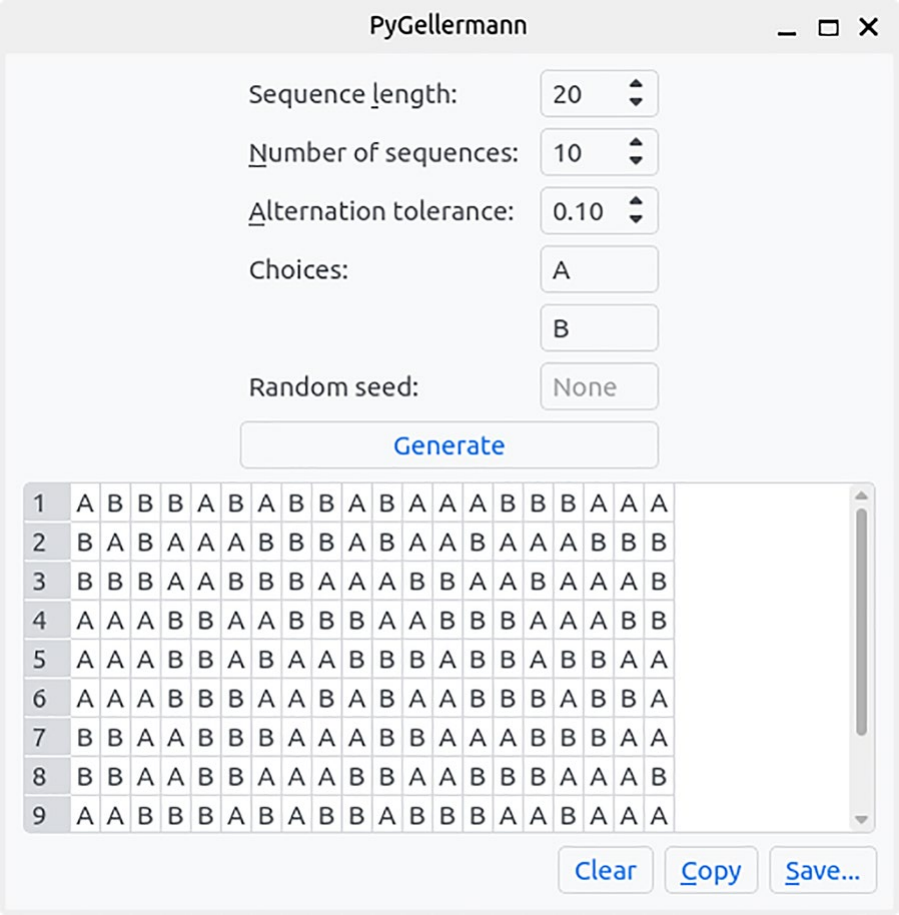
A screenshot of PyGellermann’s GUI shows the various options available to customise the generated series, as well as options to copy the generated series or save them as a table to a CSV file

From a user perspective, we provide two complementary interfaces. A graphical user interface (GUI; Fig. 3) provides a simple, straightforward way to generate any number of freshly random Gellermann series with a single mouse click. Next, the generated sequences can be saved to disk as a CSV file, which can be read by a wide range of other programs and software libraries (most notably, Microsoft Excel, Python’s *Pandas* package, or R DataFrames).

Next to the GUI, *PyGellermann* also has an application programming interface (API), which can be accessed as a Python software library. Importing the pygellermann module provides several functions which generate and return one or more Gellermann series as Python objects. As such, *PyGellermann* can be flexibly integrated as part of a larger program or application. More details can be found in the online API documentation.

More details on the installation and usage of both the graphical user interface and the Python package can be found at https://github.com/YannickJadoul/PyGellermann. *PyGellermann* has been released as open-source software under the GNU GPLv3 licence, and we invite everyone to freely use and adapt the software, as well as contribute further improvements.

Finally, we see a role for *PyGellermann* in future studies on the use of Gellermann series. After the original list of sequences [7], several arguments have been made for or against this type of randomization (e.g., [9, 11, 24]). There is a concrete opportunity for *PyGellermann* to fulfil a role in experimentally testing the merits of Gellermann series compared to fully random sequences. Such experiments can provide more empirical evidence whether the use of Gellermann series for a certain situation or species is either opportune, superfluous, or inappropriate. In such experiments, *PyGellermann* can be used to generate sets of Gellermann sequences with different “sequence length” and “alternation tolerance” parameters and test the effects on any behavioural differences with respect to fully random sequences.

## Limitations

- By definition, because the Gellermann set is a subset of all possible binary sequences, Gellermann sequences are overall ‘less random’ than a uniform distribution over all binary sequences. More precisely, if an organism could keep track of sequence regularities, it could exclude (by rote learning or even extrapolation) all those sequences which are not part of the Gellermann set.
- Previous authors have pointed out the advantages and disadvantages of using Gellermann series (e.g., [9, 11]); however, no clear consensus on a better solution has been found. Before using *PyGellermann* as an experimental tool, one should carefully consider whether Gellermann series are an appropriate randomization for the species and experiment at hand.
- As noted by previous authors [23], some original criteria detailed in [7] may be too restrictive or overspecified.
- Gellermann series are designed to ensure fair assessment of responses generated by simple strategies like perseveration or alternation (i.e., always sticking to the same answer or always switching). Our simulations and computational tests also show that Gellermann series also decently protects against more complicated response strategies such as win-stay/lose-shift or win-shift/lose-stay. However, as discussed by [24], under these simple strategies, longer streaks of correct responses may occur and reinforce these response strategies to Gellermann series.
- Reaching test criterion via binomial testing is, for a given alpha value, a function of sample size; in other words, for instance, 60% correct trials may be significant for a large sample size but not a smaller one. Studying the compound effect of this sample-size dependency and the use of Gellermann series is beyond the scope of this article, but we still warn colleagues of potential combined effects.
- The use of Gellermann series does of course in no way remove the need for a valid, well thought-out experimental setup and accompanying statistical analysis. Moreover, insofar that the experimental setting (and e.g., a participant’s motivation) allows, increasing the number of trials is the preferred way to reduce uncertainty about participants’ performance.

## Data Availability

All code is available in the GitHub YannickJadoul/PyGellermann repository, https://github.com/YannickJadoul/PyGellermann.

## Footnotes

1 Note that in the original article [7], the fifth criterion specified that the “series must offer a chance score of 50% correct from either simple or double alternation of response”. However, as noted by [23] and [25], no binary sequences of length 10 match this fifth criterion on top of the previous four. As such, we here adopt the approach proposed by [23] and generalised by [25], in which we allow a fixed margin of tolerance around 50%, determined by the user (by default, 40–60%).
